# Gemcitabine-loaded albumin nanospheres (GEM-ANPs) inhibit PANC-1 cells *in vitro* and *in vivo*

**DOI:** 10.1186/1556-276X-8-176

**Published:** 2013-04-17

**Authors:** Ji Li, Yang Di, Chen Jin, Deliang Fu, Feng Yang, Yongjian Jiang, Lie Yao, Sijie Hao, Xiaoyi Wang, Sabin Subedi, Quanxing Ni

**Affiliations:** 1 Department of Pancreatic Surgery, Huashan Hospital, Fudan University, Shanghai, 200040, China; 2 Pancreatic Disease Institute, Fudan University, Shanghai, 200040, China

**Keywords:** Gemcitabine, Albumin, Nanospheres, Antitumor

## Abstract

With the development of nanotechnology, special attention has been given to the nanomaterial application in tumor treatment. Here, a modified desolvation-cross-linking method was successfully applied to fabricate gemcitabine-loaded albumin nanospheres (GEM-ANPs), with 110 and 406 nm of mean diameter, respectively. The aim of this study was to assess the drug distribution, side effects, and antitumor activity of GEM-ANPs *in vivo*. The metabolic viability and flow cytometry analysis revealed that both GEM-ANPs, especially 406-nm GEM-ANPs, could effectively inhibit the metabolism and proliferation and promote the apoptosis of human pancreatic carcinoma (PANC-1) *in vitro*. Intravenous injection of 406-nm GEM-ANPs exhibited a significant increase of gemcitabine in the pancreas, liver, and spleen of Sprague–Dawley rats (*p* < 0.05). Moreover, no signs of toxic side effects analyzed by blood parameter changes were observed after 3 weeks of administration although a high dose (200 mg/kg) of GEM-ANPs were used. Additionally, in PANC-1-induced tumor mice, intravenous injection of 406-nm GEM-ANPs also could effectively reduce the tumor volume by comparison with free gemcitabine. With these findings, albumin nanosphere-loading approach might be efficacious to improve the antitumor activity of gemcitabine, and the efficacy is associated with the size of GEM-ANPs.

## Background

Chemotherapy is an important method of adjuvant therapy for pancreatic cancer. Gemcitabine, 2^′^,2^′^-difluoro-2^′^-deoxycytidine, remains the standard of use and has more significant clinical benefit than fluorouracil (5-FU) (clinical benefit response, 23.8% of gemcitabine treated patients vs. 4.8% of 5-FU-treated patients, *p* = 0.0022) [1,2]. However, gemcitabine has a short half-life *in vivo* and will be rapidly and extensively decomposed to inactive products in the blood, liver, kidney, and other tissues by cytidine deaminase [3]. For example, at the standard dose of 1,000 mg/m^2^, a patient’s plasma gemcitabine concentration dropped to only 0.4 μg/mL in 1 h after intravenous infusion, considerably below the 5-μg/mL optimal plasma concentration for cancer cell inhibition [4]. Thus, a larger dose is necessary, while it poses a greater risk of side effects. It has been documented that change in the formulation of gemcitabine might be a way to reduce side effects and improve the drug biopharmaceutical features [5]. For example, Paolino et al. found that gemcitabine-loaded PEGylated unilamellar liposomes could promote the concentration of the drug inside the tumor and increase the plasmatic half-life of gemcitabine [3]. Moreover, this formulation did not display any blood toxicity.

Of the various formulations available, nanospheres with a mean diameter of 10 to 1,000 nm are widely used as carriers in drug delivery systems in clinical applications [6,7]. They have some potential chemotherapeutic advantages for the treatment of tumors, including pancreatic cancer. Firstly, they can be biodegradable after intravenous injection. Secondly, owing to enhanced permeability and retention (EPR) effects, nanospheres loaded with drugs can release drugs slowly and deposit them in the target organ so that their toxicity would be enhanced in tumor tissues while reduced in normal tissues [8-10]. Furthermore, tumor cells, Kupffer cells, and mononuclear phagocyte system have higher phagocytotic rates for uptaking nanoparticles than other tissue cells. Therefore, the nanospheres loaded with drugs could be targeted to tumor, the liver, or spleen [11].

As the most abundant protein in the body, albumin is playing an increasing role as a drug carrier in the clinical setting, without hemolytic and immunogenic problems [12-14]. Previously, our research group designed a modified desolvation-cross-linking method to successfully fabricate gemcitabine-loaded albumin nanospheres (GEM-ANPs) with different sizes [15]. In this study, human pancreatic carcinoma (PANC-1) was further applied to detect the antineoplastic effects of GEM-ANPs. In particular, the *in vivo* antitumor activity of GEM-ANPs was tested in a PANC-1-induced nude mice xenograft model. Additionally, the drug distribution and toxic side effects of GEM-ANPs were also investigated.

## Methods

### Materials

Gemcitabine (hydrochloride) was purchased from Hansen Pharmaceutical Co., Ltd. (Jiangsu, China), and bovine serum albumin (BSA, ≥98%, Mw = 68,000) was purchased from Bo’ao Biological Technology Co., Ltd. (Shanghai, China). PANC-1, an ATCC human pancreatic cancer cell line, was purchased from the Shanghai Institute of Biochemistry and Cell Biology (Shanghai, China). All other solvents and chemicals were analytical grade.

### Preparation of gemcitabine-loaded albumin nanospheres

GEM-ANPs, with a mean diameter of 110 nm (110-nm GEM-ANPs) and 406 nm (406-nm GEM-ANPs), respectively, were prepared using a modified desolvation-cross-linking method according to our previous work [15]. Briefly, 10 mL of 2% BSA aqueous solution was mixed with 17 to 22 mg of gemcitabine at room temperature. The pH value of the mixed solution was adjusted to 8.0 to 9.0. An adequate amount of ethanol was added dropwise at a rate of 1 mL/min under stirring. Then the equivalent gemcitabine aqueous solution (pH 8.5) was added into the mixed solution. After stirring for 30 min, glutaraldehyde was added, and the reaction system was allowed to cross-link under stirring. The ethanol was removed by a rotary evaporator at 40°C (ZX-91, Institute of Organic Chemistry, Chinese Academy of Science, Shanghai, China). The nanospheres were centrifuged at 18,640×*g* for 20 min. Finally, the precipitation was washed with pure water three times, and the nanosphere powder could be obtained after lyophilization treatment.

In this study, 110-nm GEM-ANPs could be fabricated at pH 9.0, with an albumin/ethanol volume ratio of 1:2.5, a glutaraldehyde/albumin acid molar ratio of 1:1, and 6 h of cross-linking time. On the other hand, 406-nm GEM-ANPs could be fabricated at pH 8.0, with an albumin/ethanol volume ratio of 1:4, a glutaraldehyde/albumin acid molar ratio of 3:1, and 12 h of cross-linking time. The mean diameter, drug loading, drug encapsulation efficiency, and zeta potential were 109.7 ± 2.2 nm and 405.6 ± 3.5 nm, 11.25% and 13.40%, 82.92% and 92.56%, and −24.4 and −15.6 mV for 110-nm GEM-ANPs and 406-nm GEM-ANPs, respectively. The blank ANPs were prepared using the same procedure as that for the drug-containing nanospheres but without the addition of gemcitabine.

### Antineoplastic activity of GEM-ANPs *in vitro*

#### Cell metabolic activity assay

PANC-1 cells were cultured in RPMI 1640 supplemented with 10% fetal calf serum, 50 U penicillin/mL, and 50 μg streptomycin/mL in a humidified atmosphere with 95% O_2_ and 5% CO_2_ at 37°C. Exponentially growing cells were seeded into 96-well plates and preincubated for 24 h. Then the medium was replaced with the fresh RPMI 1640 medium containing 0.01 to 50 μg/mL of gemcitabine or GEM-ANPs or ANPs. Samples were sterilized by 60 Co radiations before exposure to cells. Cell activity after 72 h of further culture was measured by 3-(4,5-dimethylthiazol-2-yl)-2,5-diphenyl tetrazolium bromide assay (MTT) with optical density at 490 nm (OD490 nm) using a micro plate reader (EL×800, BioTek, Winooski, VT, USA) (*n* = 5). A blank control group without medication was used as control. The inhibition rate was calculated as follows:

$$\mathrm{Inhibition}\;\mathrm{rate}\;=\;\left(\mathrm{ODc}\;-\;\mathrm{ODt}\right)/\mathrm{ODc}\;\times \;100\%$$

where ODc and ODt are the OD490 nm values of the control group and the treatment group, respectively. The half maximal inhibitory concentration (IC_50_) was calculated with the Bliss method [16,17].

#### Cell cycle analysis by flow cytometry

After exposure to different samples for 72 h, PANC-1 cells were released by treatment with trypsin, washed with phosphate buffered solution (0.01 M, pH 7.4), and fixed in ice-cold 95% ethanol. After centrifugation at 252×*g* for 5 min, the cells were pretreated with 1 mL Triton X-100 and centrifuged at 252×*g* for 5 min. A further treatment with 1 mL RNase was performed at 37°C for 10 min. Then the DNA of cells was stained with 1 mL propidium iodide. Cell cycle variation after different treatment was analyzed with a FACS flow cytometer (FACS Calibur, Becton-Dickinson, Franklin Lakes, NJ, USA) using the Cell Quest software. All experiments were performed in triplicate.

### Drug distribution and toxic side effect assessment *in vivo*

#### Animals

Male Sprague–Dawley (SD) rats, 4 to 5 weeks old, (Shanghai SLAC Laboratory Animal Co., Ltd., Shanghai, China) were housed in sterilized cages and fed with autoclaved food and water *ad libitum*. Athymic nude male mice, 6 to 8 weeks old, were purchased from Shanghai SLAC Laboratory Animal Co., Ltd. and housed in a specific pathogen-free animal facility. All animal procedures were approved by the institutional animal care committee, the Science and Technology Commission of Shanghai Municipality. All guidelines met the ethical standards required by law and also complied with the guidelines for the use of experimental animals in China.

#### Drug distribution

A total of 30 clean laboratory SD rats, with an average weight of 200 g, were randomly divided into three groups as follows:

Group A: 110-nm GEM-ANPs

Group B: 406-nm GEM-ANPs

Group C: pure gemcitabine

Samples were sterilized by 60 Co radiations and dispersed into 1 mL saline before injection. After being anesthetized with 10% chloral hydrate by intraperitoneal injection (3.0 mL/kg), SD rats were injected with the solution through the femoral vein. The amount of the injection in the 110-nm GEM-ANP group, 406-nm GEM-ANP group, and gemcitabine group was converted from gemcitabine (90 mg/kg, *n* = 10). Six hours later, the animals were killed. Tissues from the pancreas, liver, spleen, heart, lung, and kidney were taken out and directly kept in liquid nitrogen. When the gemcitabine concentration was analyzed, 0.2 g tissue was taken out and homogenized with an adequate amount of physiological saline. After centrifugation at 5,000×*g* for 5 min at 4°C, 0.2 mL of the supernatant was mixed with 0.1 mL 5-bromouracil and 1 mL methanol/acetonitrile (1:9, *v*/*v*) by swirling. Then the mixed solution was kept static for 2 min and centrifuged at 5,000×*g* for 5 min at 4°C. The supernatant was flushed with nitrogen gas and resolved in the mobile phase, containing 125 μL of 0.05 mol/L ammonium acetate buffer and methanol (pH 5.7, 90:10, *v*/*v*). After centrifugation at 5,000×*g* for 5 min at 4°C, the gemcitabine content in the supernatant was determined by high-performance liquid chromatography (HPLC), with a Diamond C18 chromatographic column (5 μm, ID 4.6 × 300 mm, Anoka, MN, USA) and at a flow rate of 1 mL/min.

#### Toxic side effect assessment

Both the high-dose (200 mg/kg) and low-dose (100 mg/kg) groups were constructed, as shown in Table 1. After administration for 3 weeks, each blood sample was collected from the arteriae femoralis. Different blood parameters, including white blood count (WBC), red blood cell count (RBC), hemoglobin (Hb), alanine aminotransferase (ALT), aspartate aminotransferase (AST), creatinine (Cr), and urea (BUN), were measured using a biochemical autoanalyzer (Type 7170, Hitachi, Tokyo, Japan). The samples obtained from healthy mice were used as control.

**Table 1:** Blood parameters of SD rats treated with the different formulations for 3 weeks

**Parameters**	**Formulation (*n *= 6, *p *> 0.05)**							
	**110-nm GEM-ANPs**		**406-nm GEM-ANPs**		**Gemcitabine**		**ANPs**	**Control**
	**Normal dose**	**High dose**	**Normal dose**	**High dose**	**Normal dose**	**High dose**	**High dose**	**-**
WBC (10^9^/L)	7.3 ± 1.1	5.3 ± 2.0	6.1 ± 1.2	5.1 ± 2.2	6.1 ± 1.3	4.8 ± 2.8	8.2 ± 2.2	7.3 ± 1.9
RBC (10^12^/L)	5.6 ± 1.8	6.2 ± 1.6	6.2 ± 2.1	6.1 ± 1.1	6.5 ± 2.9	6.0 ± 2.0	6.6 ± 2.9	6.4 ± 1.2
Hb (g/L)	130.0 ± 23.0	134.0 ± 20.0	141.0 ± 14.0	138.0 ± 16.0	139.0 ± 20.0	132.0 ± 16.0	148.0 ± 23.0	143.0 ± 19.0
ALT (U/L)	44.8 ± 14.0	52.5 ± 12.9	46.0 ± 11.3	54.3 ± 12.8	51.8 ± 15.3	60.2 ± 21.9	44.7 ± 11.5	48.8 ± 13.2
AST (U/L)	109.1 ± 22.1	128.0 ± 31.8	115.5 ± 26.0	113.1 ± 26.9	129.4 ± 28.1	136.3 ± 33.4	113.3 ± 28.4	109.5 ± 25.7
Cr (mM/L)	7.1 ± 2.4	8.7 ± 3.2	6.2 ± 1.5	7.8 ± 2.07	6.1 ± 1.9	7.4 ± 2.2	4.9 ± 1.5	6.1 ± 1.6
BUN (μM/L)	41.0 ± 15.1	45.5 ± 17.3	35.4 ± 16.0	40.9 ± 19.5	36.1 ± 18.2	45.0 ± 13.7	47.2 ± 16.2	41.3 ± 18.6

### Antitumor activity *in vivo*

#### Tumor induction and drug administration

Each male nude mice (*n* = 30) was injected subcutaneously in the back skin with 0.2 mL PANC-1 cell line (1.0 × 10^8^/mL). Those mice were randomly divided into five groups (*n* = 6):

Group A: 110-nm GEM-ANPs

Group B: 406-nm GEM-ANPs

Group C: pure gemcitabine

Group D: blank ANPs

Group E: control (0.9% NS)

One week later, a tumor about 5 mm in diameter could be observed in the mice. Then five groups of mice (*n* = 6) were treated i.v. (200 μL, 120 mg/kg) with gemcitabine or GEM-ANPs containing the equivalent gemcitabine every 5 days, and a total of four treatments was performed. Control mice received 200 μL of saline, while blank mice were treated with unloaded ANPs.

#### Antitumor activity assessment

Tumor size was measured with a vernier caliper at the given intervals. Tumor volume (TV) was calculated with the following formula:

TV=16×π×a×b2

where *a* and *b* were the long and short diameter of tumor, respectively. Five weeks later, the animals were killed and weighed. Tumors were stripped and weighed. Moreover, the diameter and volume of tumors were also measured. Tumor volume inhibition rate = (Differences in mean tumor volume between the beginning and end of treatment group) / (Differences in mean tumor volume between the beginning and end of control group) × 100%; Tumor weight inhibition rate = (Differences in mean tumor weight between treatment group and control group) / (Mean tumor weight of control group) × 100%.

#### Histological analysis

The tumor tissues were carefully removed from each animal, fixed with 10% formalin, dehydrated in alcohol, and then embedded in paraffin. After sectioning and hematoxylin and eosin staining, the samples were examined to analyze the histological changes of the tissues.

#### Tumor proliferation and apoptosis analysis

The samples were stained by the method of EnVision (enhance labeled polymer system). In the microscopy vision, the background was blue or purple, and the positive products were yellow or brown. Ten consecutive cells under the ordinary optical microscope were observed, and the number of positive cells in at least 1,000 cells was counted. Tumor proliferation index (PI) was calculated as a percentage of Ki-67-positive cells.

Terminal transferase dUTP nick end labeling (TUNEL) assay is a method used to detect DNA degradation in apoptotic cells, and TUNEL kit was purchased from the Boehringer Mannheim GmbH (Mannheim, Germany). Brown particles in nucleus is determined to be the positive apoptotic cells. Ten consecutive cells were observed, and the number of positive cells in at least 1,000 cells was counted. The tumor apoptosis index (AI) was expressed as a percentage of the TUNEL-positive cells in the tumor cells.

### Statistical analysis

The number of independent replica was listed individually for each experiment. All data were expressed as mean ± standard deviation. Statistical analysis was performed with analysis of variance using SPSS 11.5 software, and *p* < 0.05 was considered to be statistically significant.

## Results

### Cytotoxicity of GEM-ANPs on PANC-1 cells *in vitro*

**Figure 1:**
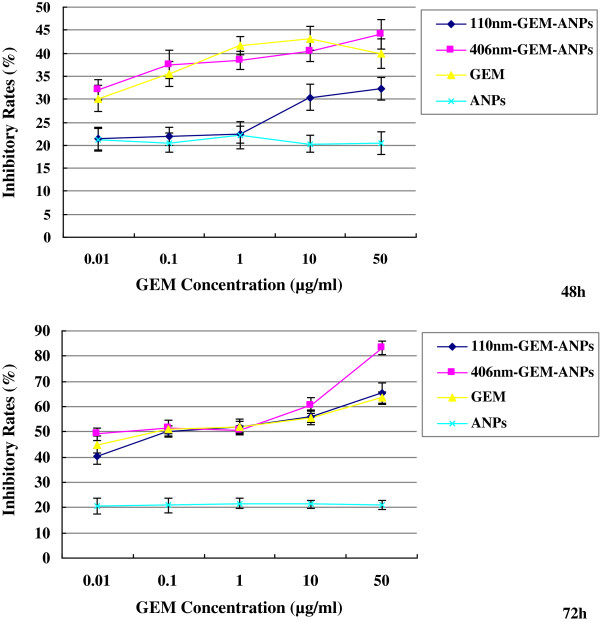
**Inhibition rate.** Gemcitabine concentration profile of 406-nm GEM-ANPs, 110-nm GEM-ANPs, gemcitabine, and ANPs on the human pancreatic cancer cell line PANC-1 after exposure for 48 and 72 h *in vitro*.

Figure 1 shows the inhibition rates of ANPs, gemcitabine, 110-nm GEM-ANPs, and 406-nm GEM-ANPs on the metabolism of PANC-1 cells measured by the MTT method, which is associated with the function of the mitochondria. It can be seen that the inhibition rate of ANPs reaches about 20% in 72 h, and the exposure time and tested concentration of ANPs have no effect on cell metabolism. Generally speaking, the inhibition effect of gemcitabine, 110-nm GEM-ANPs, and 406-nm GEM-ANPs on PANC-1 cells increases with the increase of concentration and the prolongation of the exposure time. However, 110-nm GEM-ANPs can only show a significant inhibition after 48 h of exposure when the concentration is over 10 μg/mL. With the prolongation of the exposure time, the toxicity of 110-nm GEM-ANPs obviously enhances, and 0.01 μg/mL of sample could result in a 40.25 ± 3.06% inhibition rate in 72 h. Moreover, the IC_50_ value can be calculated to be 0.10 μg/mL. Additionally, both gemcitabine and 406-nm GEM-ANPs exhibit a higher inhibition effect on PANC-1 cells in 48 h, but no significant difference between both of them can be observed. After 78 h of exposure, the IC_50_ values of gemcitabine and 406-nm GEM-ANPs reach 0.04 and 0.05 μg/mL, respectively. Especially, 406-nm GEM-ANPs display a higher inhibition rate than gemcitabine when the concentration reaches 50 μg/mL (*p* < 0.05).

The classification of cells into various phases of cell cycle was measured by flow cytometry technique, and the corresponding proliferation index and apoptosis index were calculated, as shown in Table 2. The PI cell cycle analysis reveals that cell proportion at the G0-G1 phase is significantly increased after exposure to 110-nm GEM-ANPs and 406-nm GEM-ANPs as compared with the control (*p* < 0.05), but contrary to cells at the S and G2-M phases. Both blank ANPs and gemcitabine do not show significant difference compared with the control at the proliferation index (*p* > 0.05). In addition, the AI cell cycle analysis reveals that the apoptotic cells increase from 1.8 ± 0.7% in the control group to 3.6 ± 1.5% in the 110-nm GEM-ANP group, to 6.3 ± 1.2% in the 406-nm GEM-ANP group, and to 3.7 ± 0.4% in the gemcitabine group, respectively.

**Table 2:** The proliferation and apoptosis of the pancreatic cancer cell line

**Group**	**G0-G1 (%)**	**S (%)**	**G2-M (%)**	**PI (%)**	**AI (%)**
110-nm GEM-ANPs	45.8	43.6	10.6	54.2 ± 8.7^*^	3.6 ± 1.5^*^
406-nm GEM-ANPs	44.0	48.5	7.5	56.0 ± 8.1^*^	6.3 ± 1.2^*^
Gemcitabine	35.3	46.5	18.2	64.67 ± 6.4	3.74 ± 0.4^*^
ANPs	25.9	55.4	18.8	74.11 ± 3.6	2.56 ± 0.1
Control	28.6	53.6	17.9	71.46 ± 4.8	1.78 ± 0.7

After exposure to 0.1 μg/mL of different samples for 72 h, analyzed by flow cytometry technique (*n* = 5). ^*^Significant difference compared with both control group and ANP group, *p* < 0.05.

### Biodistribution and side effect assessment of GEM-ANPs *in vivo*

Table 3 shows the gemcitabine content in different tissues after injection of gemcitabine, 110-nm GEM-ANPs, and 406-nm GEM-ANPs for 6 h, respectively, determined by HPLC. It can be seen that the gemcitabine concentration in the 406-nm GEM-ANP group is significantly increased in the liver, spleen, and pancreas (*p* < 0.05). It reaches values 5.4, 2.1, and 1.4 times higher than those in the gemcitabine group, respectively. However, no significant difference among other organs could be observed (*p* > 0.05). Table 1 showed the different blood parameters in order to assess the toxic side effects of GEM-ANPs. With respect to those observed for untreated healthy mice, both the low- and high-dose groups of 110-nm GEM-ANPs and 406-nm GEM-ANPs elicit no significant variation of rat blood parameters after 3 weeks of administration (*p* > 0.05).

**Table 3:** Gemcitabine contents (μg/g) in different organs of SD rats

**Organ**	**110-nm GEM-ANPs**	**406-nm GEM-ANPs**	**Gemcitabine**
Heart	104.9 ± 11.1	113.3 ± 18.9	117.1 ± 15.9
Liver	2.7 ± 2.5^*^	43.6 ± 13.4^*^	8.0 ± 7.2
Spleen	2.8 ± 1.9^*^	35.3 ± 7.8^*^	16.9 ± 5.1
Pancreas	101.6 ± 13.8	155.6 ± 11.8^*^	112.6 ± 5.8
Lung	8.0 ± 3.7	8.3 ± 3.6	13.9 ± 7.3
Muscle	92.8 ± 15.1	81.6 ± 11.3	84.9 ± 5.4
Kidney	105.8 ± 15.6	92.1 ± 12.9	99.7 ± 7.7

After administration of 110-nm GEM-ANPs, 406-nm GEM-ANPs, and gemcitabine for 6 h, respectively (*n* = 30). ^*^Significant difference compared with gemcitabine group, *p* < 0.05.

### Antitumor activity of GEM-ANPs *in vivo*

**Figure 2:**
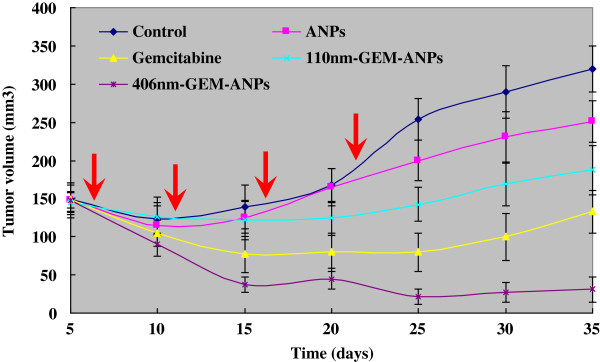
**Tumor growth curves in a PANC-1-induced nude mice xenograft model after different treatments.** Red arrows indicate the time point of administration.

**Figure 3:**
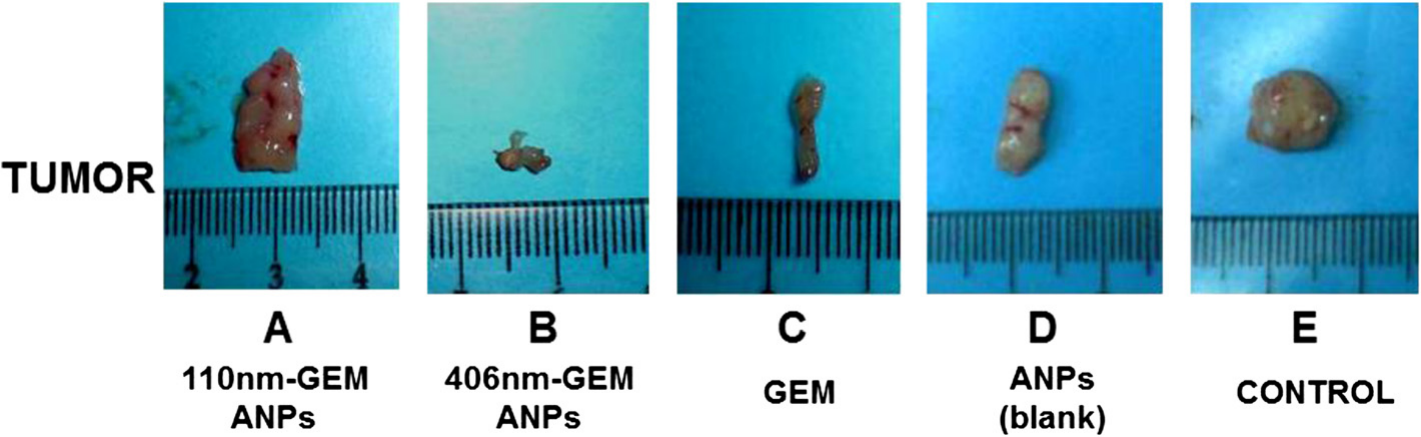
**Neoplastic mass comparison among different treatment groups.** After being excised from the PANC-1-induced nude mice tumor model following their scarification at the end of the experiments. **A** 110-nm GEM-ANPs, **B** 406-nm- GEM-ANPs, **C** gemcitabine, **D** ANPs, and **E** control.

After 5 weeks of treatment, the tumor growth curve was drawn using the checkpoint data every 5 days, as shown in Figure 2. The control group exhibits a gradual increase trend in the tumor volume, ranging from 149.4 ± 18.2 mm^3^ to 240.7 ± 37.8 mm^3^ (Figure 2). However, the tumor volume in the mice treated with 406-nm GEM-ANPs decreases gradually and varies from 148.19 ± 10.35 mm^3^ to 23.7 ± 20.1 mm^3^. Moreover, the inhibition rate of tumor volume reaches 168.8% (Table 4). Besides, both gemcitabine and 110-nm GEM-ANPs can also inhibit the increase of tumor volume, and the inhibition rate reaches 109.9% and 75.1%, respectively (Table 4). However, the tumor volume shows an increase trend after discontinuation of 110-nm GEM-ANPs or gemcitabine (Figure 2). The weight of the collected tumor masses confirms these findings. In fact, masses of 0.175, 0.090, and 0.166 g were observed in the case of 110-nm GEM-ANPs, 406-nm GEM-ANPs, and gemcitabine treatment, respectively, while control animals and ANPs show tumoral masses of 0.291 and 0.245 g, respectively (Table 4 and Figure 3). Besides, the reduction in tumor blood supply could be seen in the 406-nm GEM-ANP group, while they are relatively rich in the gemcitabine group and abundant in the ANP group and control group (Figure 3).

**Table 4:** The inhibition rate of GEM-ANPs on tumor growth in the PANC-1-induced nude mice tumor model

**Group**	**Tumor volume (mm**^**3**^**)**		**Volume change, Δ*V *(mm**^**3**^**)**	**Inhibitory rate of volume**^**a **^**(%)**	**Tumor weight**^**b **^**(g)**	**Inhibitory rate of weight**^**c **^**(%)**
	**5 days**	**35 days**				
110-nm GEM-ANPs	144.9 ± 12.2	187.3 ± 32.4	42.4	75.1	0.175	39.9
406-nm GEM-ANPs	148.2 ± 10.4	31.0 ± 16.1	−117.2	168.8^*^	0.090^*^	69.1^*^
Gemcitabine	149.64 ± 20.35	132.80 ± 28.2	−16.8	109.9	0.166	43.0
ANPs	147.6 ± 22.7	250.6 ± 27.2	103.0	39.6	0.245	15.81
Control	149.4 ± 18.2	319.9 ± 30.3	170.5	0.0	0.291	0.0

*n* = 30. ^a^Inhibition rate of tumor volume = (Differences in mean tumor volume between the beginning and end of treatment group) / (differences in mean tumor volume between the begin and end of control group) × 100%. ^b^The tumor weight was measured at 35 days after administration. ^c^Inhibition rate of tumor weight = (Differences in mean tumor weight between treatment group and control group) / (Mean tumor weight of control group) × 100%. ^*^Significant difference compared with gemcitabine group, *p* < 0.05.

**Figure 4:**
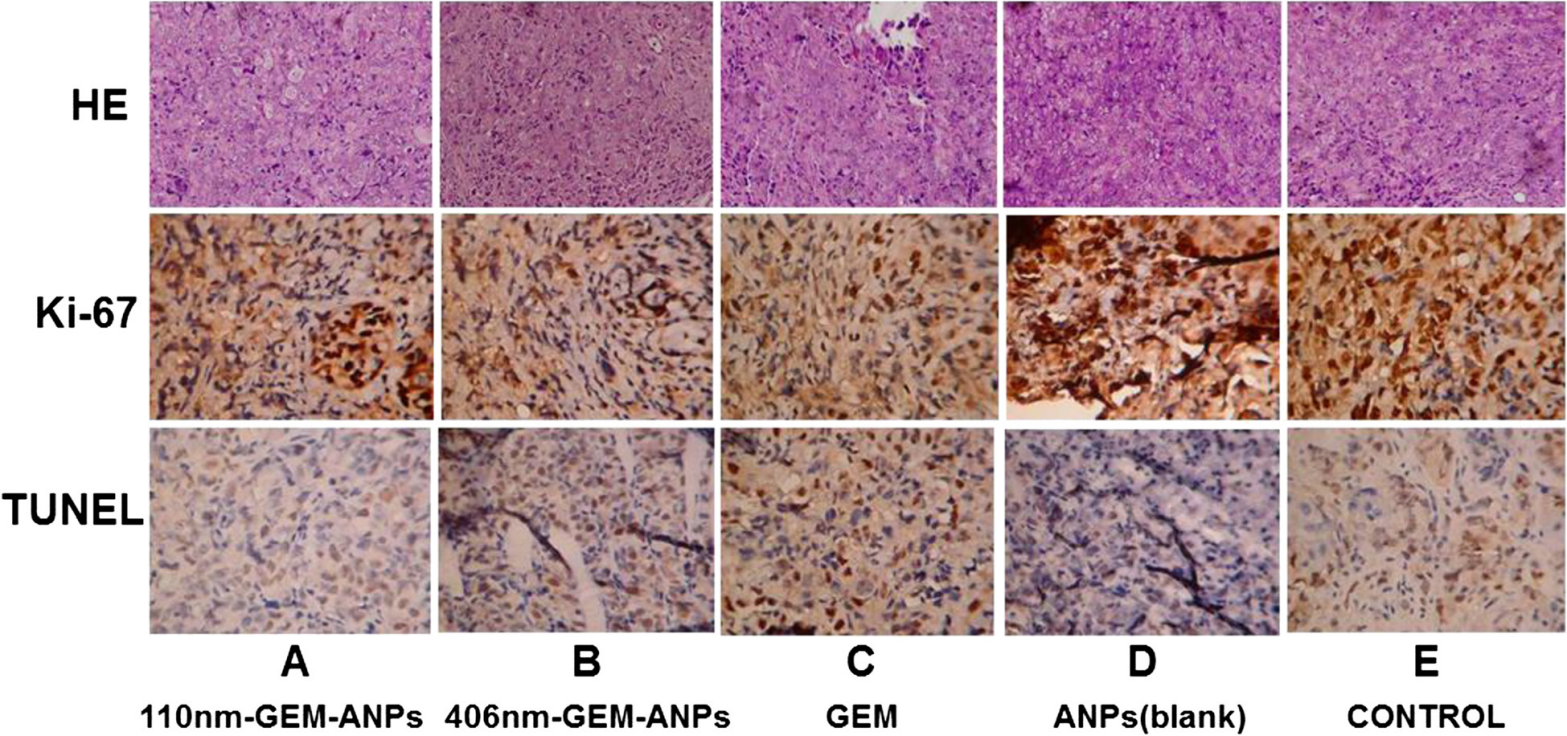
**Histological analysis of neoplastic masses by H & E staining, Ki-67 protein, and TUNEL assay after being excised from the PANC-1-induced nude mice tumor model following their scarification at the end of the experiments. A** 110nm-GEM-ANPs, **B** 406-nm-GEM-ANPs, **C** gemcitabine, **D** ANPs and **E** control.

Histological analysis of tumor masses after various treatments for 5 weeks was performed by H & E staining; the proliferation and apoptosis of tumor cells were also determined by immunohistochemical assay on Ki-67 protein and TUNEL assay, as shown in Figure 4. H & E staining confirms that the tumor cell proliferation and division are more active in the control group than in other groups. In addition, Ki-67 protein immunohistochemical assay indicates that the proliferation index of tumor cells in 110-nm GEM-ANP (36.4 ± 8.1%), 406-nm GEM-ANP (25.6 ± 5.7%), and gemcitabine (38.4 ± 9.4%) groups are lower than that in the blank ANP and control group, with significant difference (*p* < 0.05). At the same time, TUNEL assay reveals that the apoptotic index of tumor cells in the 406-nm GEM-ANP (38.5 ± 17.2%) group is significantly higher than that in the 110-nm GEM-ANP (33.6 ± 11.2) and gemcitabine (32.2 ± 9.7%) groups (Figure 4).

## Discussion

As one of the most lethal cancers, pancreatic cancer is still a frequently occurring disease and remains a therapeutic challenge to humans [18,19]. Although gemcitabine is a currently and widely used drug in the therapy of pancreatic cancer, various approaches, such as drug delivery system, have to be tried to prolong the plasma half-life of gemcitabine and enhance its bioavailability [20,21]. As the typical examples, liposome and carbon nanotube have been a success in delivering cancer drugs for pancreatic cancer treatment in recent animal and preclinical trials [19,22]. Nowadays, a novel carrier system allowing for lower toxic side effects and higher tumor-targeting efficiencies is emphasized, while the high biosafety of the carrier system is also prerequisite [8,10,23]. In our previous work, BSA was introduced to act as a drug carrier for gemcitabine loading [15]. We found that GEM-ANPs could result in a sustained release and improved antitumor activity *in vitro* of gemcitabine. Here, we further exposed human pancreatic carcinoma (PANC-1) to GEM-ANPs and studied cell responses *in vitro* by cell viability analysis and flow cytometry technique. The loading of gemcitabine on albumin did not reduce the inhibition effect of gemcitabine on PANC-1 metabolism. Moreover, GEM-ANPs with bigger size could even enhance the killing efficacy of gemcitabine in pancreatic carcinoma (Figure 1). GEM-ANPs showed their cell cycle inhibitory property, in the order of 406-nm GEM-ANPs > 110-nm GEM-ANPs > gemcitabine. The higher antiproliferative activity of 406-nm GEM-ANPs could be attributed to the S phase arrest during cell cycle progression (Table 2).

Besides the shorter half-life, the toxic side effects, like increased liver enzymes and leukopenia, have also limited the applications of gemcitabine [24]. Therefore, the blood parameters of rats treated with GEM-ANPs were investigated to assess the reduction effect of albumin loading on gemcitabine toxic side effects. Since the blank nanoparticles could interfere with the growth of cells *in vitro*, the US Pharmacopoeia limits cell inhibition as no more than 50% for safety [25]. The present study revealed that no significant difference between the ANP treatment group and control group was observed in WBC, RBC, and other parameters of hepatonephric functions, suggesting a satisfactory biocompatibility (Table 1). What was more important was that the high-dose treatment with GEM-ANPs, especially 406-nm GEM-ANPs, could reduce the side effects of gemcitabine (Table 1). In fact, gemcitabine concentration and treatment period were insufficient to induce a relevant blood toxicity in the present study [26]. Our results also demonstrated that gemcitabine loading on 406-nm GEM-ANPs significantly increased the gemcitabine content in the pancreas, liver, and spleen of SD rats compared with the gemcitabine treatment group, but contrary to 110-nm GEM-ANPs (*p* < 0.05) (Table 3). It is well known that nanospheres are easily taken up by cells of the mononuclear phagocyte system, primarily those located in the reticuloendothelial system-rich organs, such as the liver and spleen [27]. Furthermore, phagocytosis will gradually increase as the size is more than 200 nm [28]. Consequently, it might be one of the reasonable mechanisms for the targeting effect of 406-nm GEM-ANPs *in vivo*[29]. That was to say, 406-nm GEM-ANPs would enhance the curative effect of gemcitabine in pancreatic cancer. Particularly, literatures have reported that the microvascular permeability of most normal tissues was generally less than 50 nm, but ten times higher in tumor tissues and usually more than 500 nm. For example, Hobbs et al. found that the microvascular permeability of rat hepatoma, fibrosarcoma, and human colon cancer animal model reach 380 to 550, 550 to 780 and 380 to 550 nm, respectively [30]. Yuan et al. also found that the maximum diameter of microvascular permeability in human colon cancer is between 400 and 600 nm [31]. In addition, Desai [32] and Cortes and Saura [33] found that albumin nanoparticles could increase albumin receptor, 60-kDa glycoprotein (gp60)-mediated transcytosis, through microvessel endothelial cells in angiogenic tumor vasculature and targets the albumin-binding protein SPARC, which subsequently increased intratumoral accumulation. Therefore, a relatively high antitumor activity of 406-nm GEM-ANPs could be expected due to the passive targeting by EPR effect and gp60-mediated transcytosis [8-10,23,32,33]. Here, the antitumor effects of GEM-ANPs were assessed *in vivo* using the implanted tumor model of nude mice. We found that the antitumor effect of 406-nm GEM-ANPs was greatest (Figures 2 and 3), with 168.8% inhibitory rate compared to the control. Finally, the slow release of gemcitabine from 406-nm GEM-ANPs could also prolong the drug action, and it might be another possible reason for the higher antitumor activity of GEM-ANPs.

## Conclusions

GEM-ANPs with different sizes had been prepared by the modified desolvation-cross-linking method. Their biodistribution, toxic side effects, and *in vitro* and *in vivo* antitumor activity were studied. The following conclusions can be drawn from the study described here:

(1) GEM-ANPs showed significant inhibition effects on human pancreatic carcinoma, but the inhibition rate was size dependent.

(2) The suitable size of 406-nm GEM-ANPs resulted in a higher gemcitabine content in the pancreas, liver, and spleen of SD rats and a lower blood toxicity through a passive targeting model.

(3) A more efficient antitumor activity was demonstrated in a pancreatic cancer xenograft model for 406-nm GEM-ANPs with respect to that of free gemcitabine. Therefore, the orthotopic model for pancreatic cancer remains to be examined in our future work.

## Abbreviations

5-FU: fluorouracil; AI: apoptosis index; ALT: alanine aminotransferase; AST: aspartate aminotransferase; BSA: bovine serum albumin; BUN: urea; Cr: creatinine; EPR: enhanced permeability and retention; GEM-ANP: gemcitabine-loaded albumin nanosphere; H & E: hematoxylin and eosin; Hb: hemoglobin; HPLC: high-performance liquid chromatography; MTT: 3-(4,5-dimethylthiazol-2-yl)-2,5-diphenyl tetrazolium bromide assay; PANC-1: human pancreatic carcinoma; PI: proliferation index; RBC: red blood cell count; SD: Sprague–Dawley; TUNEL: transferase dUTP nick end labeling; TV: tumor volume; WBC: white blood count.

## Competing interests

The authors declare that they have no competing interests.

## Authors’ contributions

JL carried out the antitumor activity *in vivo*, performed all the experimental analysis, and drafted the manuscript. CJ carried out the experimental design. Both DF and YD fabricated the gemcitabine-loaded albumin nanospheres. FY, YJ, and LY studied the antineoplastic activity of GEM-ANPs *in vitro*. SH, XW, SS, and QN performed the drug distribution and toxic side effect assessment *in vivo* on both nanospheres. All authors read and approved the final manuscript.
